# TFP5-Mediated CDK5 Activity Inhibition Improves Diabetic Nephropathy *via* NGF/Sirt1 Regulating Axis

**DOI:** 10.3389/fcell.2022.829067

**Published:** 2022-07-07

**Authors:** Shi-Lu Cao, Hong-Yan Luo, Yong-Cai Gao, Xiao-Mei Lan, Shun-Yao Liu, Bo Li, Li Bao, Jing E., Danna Ma, Guo-Qing Zhang, Li-Rong Yang, Xi Bao, Ya-Li Zheng

**Affiliations:** ^1^ Department of Nephrology, Ningxia Medical University Affiliated People’s Hospital of Autonomous Region of Yinchuan, Yinchuan, China; ^2^ The Third Clinical Medical College of Ningxia Medical University, Yinchuan, China; ^3^ Department of Geriatrics, Ningxia Medical University Affiliated People’s Hospital of Autonomous Region of Yinchuan, Yinchuan, China; ^4^ Dialysis Department of Nephrology Hospital, The First Affiliated Hospital of Xi’an Jiaotong University, Xi’an, China; ^5^ Department of Nephrology, The Second Affiliated Hospital of Xi’an Jiaotong University, Xi’an, China

**Keywords:** TFP5, diabetic nephropathy, CDK5, NGF, Sirt1

## Abstract

Diabetic nephropathy (DN) is one of the leading causes of chronic kidney disease (CKD), during which hyperglycemia is composed of the major force for the deterioration to end-stage renal disease (ESRD). However, the underlying mechanism triggering the effect of hyperglycemia on DN is not very clear and the clinically available drug for hyperglycemia-induced DN is in need of urgent development. Here, we found that high glucose (HG) increased the activity of cyclin-dependent kinase 5 (CDK5) dependent on P35/25 and which upregulated the oxidative stress and apoptosis of mouse podocytes (MPC-5). TFP5, a 25-amino acid peptide inhibiting CDK5 activity, decreased the secretion of inflammation cytokines in serum and kidney, and effectively protected the kidney function in *db/db* mouse from hyperglycemia-induced kidney injuries. In addition, TFP5 treatment decreased HG-induced oxidative stress and cell apoptosis in MPC-5 cells and kidney tissue of *db/db* mouse. The principal component analysis (PCA) of RNA-seq data showed that MPC-5 cell cultured under HG, was well discriminated from that under low glucose (LG) conditions, indicating the profound influence of HG on the properties of podocytes. Furthermore, we found that HG significantly decreased the level of NGF and Sirt1, both of which correlated with CDK5 activity. Furthermore, knockdown of NGF was correlated with the decreased expression of Sirt1 while NGF overexpression leads to upregulated Sirt1 and decreased oxidative stress and apoptosis in MPC-5 cells, indicating the positive regulation between NGF and Sirt1 in podocytes. Finally, we found that K252a, an inhibitor of NGF treatment could undermine the protective role of TFP5 on hyperglycemia-induced DN in *db/db* mouse model. In conclusion, the CDK5-NGF/Sirt1 regulating axis may be the novel pathway to prevent DN progression and TFP5 may be a promising compound to improved hyperglycemia induced DN.

## Introduction

Chronic kidney disease (CKD) is a prevalent and irreversible syndrome of kidney disease (Ammirati, 2020). Clinically, CKD patients present a reduced glomerular filtration rate (GFR), increased urinary albumin excretion, and creatinine (Vega and Huidobro E., 2019). Usually, the patient was diagnosed as CKD when GFR <60 ml/min/1.73 m^2^, and the content of albumin was >30 mg in the 24 h urine, or isolated urine sample, rectified by urinary creatinine (Ammirati, 2020).

CKD often results in a sustained damage of renal parenchyma, and finally promotes deterioration of renal function (Akchurin, 2019). Among the pathogenic factors, diabetic nephropathy (DN) is one of the primary causes of end-stage renal disease (ESRD) world-wide (Lo et al., 2018). Fifty percent of type 2 diabetes (T2D) patients and 33% of type 1 diabetes (T1D patients) finally develop CKD (Thomas et al., 2015). The different morbidity may result from different causes, such as obesity, intrarenal vascular disease, atherosclerosis, or renal ischemia in T2D (Anders et al., 2018). CKD is correlated with the dysfunction of glomerular basement membrane composed of fenestrated endothelial cells and interdigitating cell derived from podocytes. The filtration barrier exerts its function by allowing just small molecules that are without a high negative charge to pass through (Jefferson et al., 2008). Evidence showed that excessive podocyte damage will eventually result in extensive podocyte loss, and the number of podocytes exhibited a gradual decrease as the disease deteriorated. Podocytes lacked the ability to renew, and excessive loss of podocytes will finally leads to large-scale replacement of glomerular basement membrane by the parietal epithelial cells and the Bowman capsule, which accelerates the progression segmental glomerulosclerosis (Nakamura et al., 2000; Langham et al., 2002). Therefore, the intact podocytes are necessary for the renal function in diabetic kidney disease. A number of studies showed that high intracellular glucose level could produces advanced glycation end products which could result in the expression of transforming growth factor (TGFβ1) in podocytes, promoting apoptosis and GBM thickening by stimulating extracellular matrix production (Nowotny et al., 2015; Rabbani and Thornalley, 2018). Furthermore, TGFβ inhibited the expression of integrin in podocytes, leading to podocyte detachment and podocytopenia under hyperglycemia. Except for podocytes, the increased oxidative stress and inflammation caused by hyperglycemia were also reported for its contribution to DN (Elmarakby and Sullivan, 2012; Yaribeygi et al., 2019).

CDK5 is a proline-directed Ser/Thr protein kinase that plays an important role in many cellular functions including cell motility and survival which could be activated by binding to regulatory subunit, p35, p39, or p25 (a proteolytic fragment of p35) (Lew et al., 1994). CDK5 phosphorylation regulates the function of various regulatory proteins, including members of the Bcl-2 family of pro- and anti-apoptotic proteins. Previous evidence showed that p35 and p39 are thought to be necessary and sufficient for both CDK5 activities (Ko et al., 2001). CDK5 activity can also be inhibited by the purine-based compounds Olomoucine and Roscovitine (Vesely et al., 1994; Meijer et al., 1997). However, neither of the two compounds are selective inhibitors of CDK5. For example, CDK1/2/5 and Erk1/2 could be inhibited by Olomoucine and Roscovitine, and Roscovitine could also inhibit pyridoxal kinase (Meijer et al., 1997; Knockaert et al., 2002; Bach et al., 2005). Currently, Liebl et al. (2011) are developing more specific CDK5 inhibitors. Furthermore, diabetes-associated hyperglycemia increased the CDK5 levels (Cai et al., 2020). Recent studies suggest that CDK5 plays crucial roles in physiological functions in glucose-stimulated insulin secretion in pancreatic cells, indicating that CDK5 might be a potential drug target for diabetes mellitus and subsequent DN progression (Wei and Tomizawa, 2007). Therefore, specific CDK5 inhibitor and the effect of CDK5 on DN is urgently required to be explored.

Podocytes are highly specialized cells with a key role in kidney physiology. Destruction of their structure caused by injury could result in severe renal diseases. Previous study reported the capability of podocytes to produce and secrete the nerve growth factor (NGF) in an *in vitro* model (Caroleo et al., 2015). Studies have found that the increased secretion of NGF in a high glucose (HG) environment can protect nerve cells and pancreatic islet *β* cells from HG-induced oxidative stress and inflammatory factors damage, confirming that NGF is a new biological target for podocyte regulation (Guo et al., 2011; Caroleo et al., 2015; Skaper, 2017). However, the role of NGF in diabetes-induced CKD is not very clear. In diabetics, there was an early length-dependent dysfunction of small-diameter sensory fibers, with depletion of skin NGF and the sensory neuropeptide substance P (Anand, 1996).

Sirt1 has been reported to affect oxidative stress and apoptosis *via* deacetylation of its substrates (Yu and Auwerx, 2010). Intriguingly, the inhibition of Sirt1 with pharmacological agents leads to an elevation of ROS levels and NOX production (Wosniak et al., 2009; Benard et al., 2014). It was demonstrated that Sirt1 agonist SRT1720 upregulated eNOs and superoxide dismutase (SOD) levels to ameliorate endothelial oxidative stress (Chen et al., 2012). Previous studies showed that HG stress triggers the initial changes in proximal tubules and subsequent epigenetically irreversible glomerular damages which could be rescued by proximal tubular Sirt1 overexpression. Reduction of Sirt1 expression in proximal tubules leads to the decrease of glomerular Sirt1, and affects Sirt1 expression in podocytes (Hasegawa et al., 2016). Studies on diabetic animals had shown overexpression of Sirt1 in both podocytes and renal tubular cells attenuated proteinuria and kidney injury in the animal model of diabetic nephropathy through deacetylation of NF-кB, Smad3, FOXO, and p53 (Ji et al., 2021).

Furthermore Sirt1 is one of the substrates of CDK5 (Bai et al., 2012), which plays an important protective role in cell differentiation, survival, and anti-oxidation in diabetic nephropathy (Dong et al., 2014; Kong et al., 2015). Especially in a HG environment, CDK5 has a protective effect on the structure, function, and apoptosis of podocytes (Nakatani and Inagi, 2016; Chuang et al., 2017; Rogacka et al., 2018). The hyperphosphorylation of Sirt1 by CDK5 in HG environment makes it lose the above effect (Bai et al., 2014). The latest study revealed that the NGF/Sirt1 axis may become a new therapeutic target for intervening cell damage (Tsai et al., 2018). Therefore, we propose the scientific hypothesis: The CDK5 activity in diabetic nephropathy causes podocyte injury and apoptosis by regulating the NGF/Sirt1 axis and oxidative stress inflammatory factors in podocytes.

Previous study has reported a 24-aa peptide (termed P5), which has shown the ability to inhibit CDK5/p25 activity in transfected human embryonic kidney 293 cells. Thereafter, TFP5 (modified as P5) was designed to penetrate the blood–brain barrier after intraperitoneal injections, which inhibited abnormal Cdk5/p25 hyperactivity and significantly rescued AD pathology in AD model mice (Shukla et al., 2013). In this study, we found that TFP5, a specific inhibitor of CDK5, efficiently protected the kidney function from the damage of diabetes. TFP5 promoted the survival of podocytes by inhibiting apoptosis and oxidation caused by hyperglycemia in *db/db* mice. TFP5-mediated CDK5 inhibition upregulated Sirt1 expression, and NGF was necessary for the regulating axis. In conclusion, TFP5 treatment protects the kidney from hyperglycemia-induced damage by CDK5-NGF-Sirt1 regulation axis, and NGF and Sirt1 may be the novel target for DN.

## Materials and Methods

### Animal

The male C57BLKS/J *db/db* diabetic mice at the age of 7 weeks and age-matched *db/m* mice were purchased from Southern Model Animal Co., Ltd. (Nanjing, China). All mice were raised in a SPF IVC cage with a 12 h light–dark cycle at a temperature of 25°C. Before treatment, all mice were acclimatized to the raising environment for 1 week, and the glucose level and urinary albumin level were measured and all mice were randomly divided into different groups. The mice were administrated with 150 μl TFP5 peptide (200 μM) or 150 μl scramble peptide (200 μM) by intraperitoneal injection every 3 days. Totally, the mice were treated for 16 times (from week 8 to week 16). All the experimental procedures were approved by the Institutional Animal Care and Use Committee of Ningxia Medical University (NO. 2018-026).

### Biochemistry Analysis

The level of fasting blood glucose of all mice was monitored 4 h post feeding by using blood glucose meter (Roche, Basel, Switzerland). Urinary albumin excretion was detected by the enzyme-linked immunosorbent assay (Bethyl Laboratories, Montgomery, United States) according to the manufacturer’s instructions.

### Plasmids and Cell Lines

MPC-5 cell line was purchased from ATCC. NGF overexpressed MPC-5 cells were constructed by pHBLV-CMV-MCS plasmid purchased from Hanheng biology. SiRNAs of NGF were purchased from Hanheng biology.

### Antibodies and Inhibitors

Antibodies for P35/P25 (#2680S, Clone: C64B10; Cell signaling technology), CDK5 (#14145; Clone: D1F7M; Cell signaling technology). Anti-NGF, (#ab52918; clone: EP1320Y; abcam). Anti-Sirt1 (#ab189494; clone: EPR18239; abcam). Anti-Cleaved Caspase-3 (#ab32042: clone: E83-77, abcam). Anti-Sirt1 (#ab76039, EPR2849Y, abcam). K252a (MedChemExpress; CAS No.: 99533-80-9). TFP5 and scrambled peptide (SCB) were produced by Nanjing Peptide Industry Biotechnology Co., LTD. (Sequences: TFP5, FITCGGGKEAFWDRCLSVINLMSSKMLQINAYARAARRAARR; SCB: FITCGGGGGGFWDRCLSGKGKMSSKGGGINAYARAARRAARR). SCB peptide was a designed peptide without specific effect on physiological function as we identified previously.

### Cell Culture

Immortalized mouse podocytes MPC-5 were cultured in Dulbecco’s modified Eagle medium (DMEM) composed of 10% fetal bovine serum, 2 mmol/L glutamine, 1 mmol/L sodium pyruvate, 1% penicillin and streptomycin in 37°C with 5% carbon dioxide (CO2) incubator and 95% air. The concentration of glucose in LG and HG mediums, respectively, were 1 g/L and 4.5 g/L.

### RNA Extraction and Real-Time PCR

In cell lines, the total RNA was extracted by TRIzol reagent according to established protocol. Briefly, 2 × 10^6^ cells were washed by 5 ml ice-cold PBS. 1 ml TRIzol was added and sufficiently pipetted with the cell pellet. 200 μl Chloroform was added and immediately shaken for 45 s, and then, the samples were centrifuged at 12,000 g for 15 min at 4°C. The top layer was collected into RNase free tube, and equal volume of isopropanol were added and mixed sufficiently. The samples were centrifuged at 12,000 g for 50 min. The RNA pellet was washed twice with RNase-free 75% ethanol. RNA was suspended in diethyl pyrocarbonate-treated (DEPC) water. For tissue, a 0.2 g tissue was cut off from the frozen sample, and was homogenized in liquid nitrogen. The sample was then crushed with a mortar, and the RNA extraction was continued as that for cell line. 1 μg mRNA was used for cDNA synthesis with PrimeScript RT reagent Kit (Takara, Cat. No., RR037A). Quantitative-PCR was performed in triplicates with SYBR Green Master Mix (Applied Biosystems, Carlsbad, CA, United States), with ABI PRISM 7500 facility. The expression of target genes was normalized to GAPDH expression. The 2^−ΔCT^ method was used to calculate the relative expression of targeted genes. All experiments were performed in triplicate. Primer sequences were as follows: NGF-F: 5′-CCA​GTG​AAA​TTA​GGC​TCC​CTG-3′, NGF-R: 5′-CCT​TGG​CAA​AAC​CTT​TAT​TGG​G-3′; CDK5-F: 5′-CTG​TCC​CTA​TCC​CCC​AGC​TAT-3′, CDK5-R: 5′-GGC​AGC​ACC​GAG​ATG​ATG​G-3′; Sirt1-F: 5′-TGA​TTG​GCA​CCG​ATC​CTC​G-3′, Sirt1-R: 5′-CCA​CAG​CGT​CAT​ATC​ATC​CAG-3′; Caspase 3-F: 5′-TGG​TGA​TGA​AGG​GGT​CAT​TTA​TG-3′, Caspase 3-F: 5′-TTC​GGC​TTT​CCA​GTC​AGA​CTC-3′; Bax-F: 5′-AGA​CAG​GGG​CCT​TTT​TGC​TAC-3′, Bax-R: 5′-AAT​TCG​CCG​GAG​ACA​CTC​G-3′; Bcl-2-F:5′-GAGAGCGTCAACAGGGAGATG-3′, Bcl-2-R: 5′-CCA​GCC​TCC​GTT​ATC​CTG​GA-3′.

### RNA Sequence

MPC-5 cells cultured under LG and HG were collected. RNA was extracted and cDNA was then produced by Takara’s Reverse Transcriptase kit. Then, the amplification of all cDNA samples was performed with the KAPA Library Quantification kit for 22–25 cycles. The samples were sequenced on the computer to obtain image files, which were converted by the software of the sequencing platform to generate the raw data of FASTQ. We counted the raw data of each sample separately, used Cutadapt to remove the linker at the 3′ end, and removed the Reads with an average quality score lower than Q20. Then, we assessed sequencing data quality by taking base quality distribution, base content distribution, and reads average quality distribution. Finally, the amplified cDNA library was sequenced and analyzed. The FPKM (fragments per kilobase of transcript per M) was calculated and used to estimate the abundance of gene expression.

### Flow Cytometry

We detected the expression of CDK5 (Second antibody goat anti-mouse Alexa Fluor 488) on MPC-5 cells under HG and TFP5 treatment. Annexin V (AV) and propidium Iodide (PI) were used to detect the apoptosis by flow cytometry. Briefly, MPC-5 cells were washed with ice cold PBS twice. We then added PI (10 μl) and AV-FITC (AV-FITC, 10 μl) to a cell suspension of 100 μl and incubated them at room temperature in the dark for 15 min. For CDK5 staining, 2 × 10^6^ MPC-5 cells were resuspended by staining buffer, and 5 μl antibodies were added to the sample. The cells were incubated at 4°C for 30 min in darkness. Then, 5 μl goat anti-mouse Alexa Fluor 488 was added and incubated at 4°C for another 30 min in darkness. Finally, the cells were washed with PBS twice and analyzed with BD Fortessa equipment. Finally, the data was analyzed by FlowJo software (V10).

### ROS Detection

Intracellular ROS production was measured using 2′,7′-dichlorodihydrofluorescein diacetate (DCFH-DA) (Beyotime, Shanghai, China). Seed fibroblasts were kept in 60 mm dishes. After incubation, cells were harvested using trypsin-EDTA solution. The cell suspension was then centrifuged at room temperature for 5 min and the supernatant was removed. The fluorescence intensity of DCFH-DA was measured and calculated using a flow cytometer (Fortessa, BD).

### H_2_O_2_ Detection

The cells were collected in a centrifuge tube and the supernatant was discarded. 100–200 μl hydrogen peroxide detection lysis solution was added into per 1 × 10^7^ cells and then fully homogenized to disrupt and lyse the cells. Centrifuge was at about 12,000 g for 3–5 min at 4°C, and the supernatant was taken for subsequent determination by using hydrogen peroxide test kit (Beyotime, cata# S0038).

### Evaluation of CDK5 Activity

CDK5 activity were measured according to a previous study (Zheng et al., 2016). Briefly, the CDK5 complex was obtained by immunoprecipitation from tissue lysates. Then, the extracted CDK5 complex was incubated with histone H1 for 1 h at a temperature of 30°C. After the incubation was completed, a moderate volume of SDS loading buffer was added and then boiled for 5 min. The phosphorylation levels of histone H1 were regarded as a reflection of CDK5 kinase activity.

### Immunocytochemistry

The mouse kidney was harvested and fixed with 4% paraformaldehyde overnight. The fixed tissues were cut into 4 μm sections. The sections were warmed at 80°C for 2 h and immediately dewaxed in xylene. Then, the sections were serially rehydrated with 100% ethanol, 85% ethanol, 75% ethanol, and 50% ethanol. The samples were incubated by target primary antibody at 4°C for 12 h. After the antigen retrieval with pH = 6.0 sodium citrate buffer in microwave oven, the sections were incubated in 3% H_2_O_2_ (diluted with methanol) to block endogenous peroxidases and was blocked in normal goat serum. The sections were then incubated with CDK5, Sirt1, NGF, P35/25 overnight at 4°C, followed by incubation with an instant biotinylated corresponding lgG antibodies. Finally, the sections were incubated with SABC reagents and staining with DAB and hematoxylin. Finally, sections were imaged under a microscope and six different fields were randomly captured to evaluate the intensity of staining. Expression of the CDK5, NGF, Sirt1, and P35/25 proteins was assessed by the positive signal for the staining intensity (0, negative; 1, light; 2, moderate; 3, strong). The representative pictures are shown in the figures.

### Histological Evaluation Score

The fresh renal tissue specimens isolated from mice were fixed and cut in 4 μm, and was stained with HE according to standard procedures. Three sections per kidney were evaluated under a light microscope. All samples were independently calculated by at least two experienced renal pathologists in a blinded method. Pathological scoring (0–5) was used to assess the degree of glomerulosclerosis scores. Eight random areas were selected. Pathological scoring ranged from 0 to 5 points of injury area (IA): 0, normal; 1, IA < 10%; 2, 10% < IA < 25%; 3, 25% < IA < 50%; 4, 50% < IA < 75%; 5, IA > 75%.

### Statistical Analysis

The unpaired Student’s t-test (two-sided) was used to calculate the statistical difference of two groups, and two-way ANOVA analysis was performed for multiple comparisons. All results in figures are the representative results of at least three independent experiments. The values in graphs are exhibited as mean ± SD. Analyses and figures were obtained by using GraphPad 7.0 software.

## Results

### HG Increased the Expression and Activity of CDK5 and in Mouse Podocytes

**FIGURE 1:**
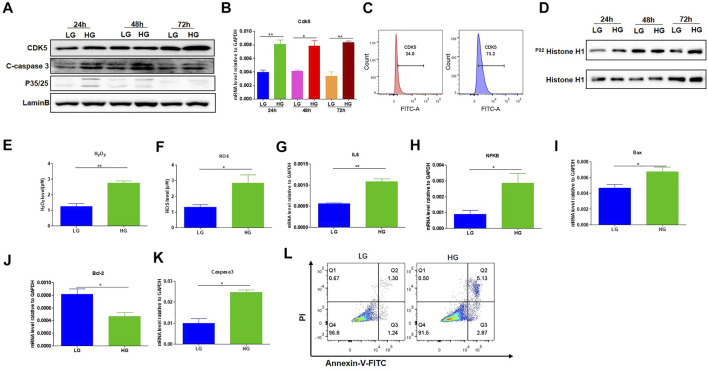
HG-promoted oxidative stress-induced apoptosis in podocytes. **(A)** The effect of HG treatment on protein level of CDK5, p35/25 and apoptosis-related cleaved caspase-3 in MPC-5 cell lines. The cells were treated for 24, 48, and 72 h with LG and HG conditions, respectively. LaminB was regarded as the loading control. **(B)** The change of mRNA of CDK5 after 24, 48, and 72 h treatment with LG and HG in MPC-5 cells. **(C)** The detection of CDK5 expression in MPC-5 cells after HG treatment by flow cytometry. The cells were treated for 24 h under HG and LG conditions, and the goat anti-rabbit 488 secondary antibody was used. **(D)** The effect of HG treatment on the activity of CDK5 at different timepoints. Cdk5 was harvested by C-8 antibody by immunoprecipitation. The immunoprecipitate was then subjected to *in vitro* histone H1 kinase assays. **(E,F)** The effect of HG treatment on the level of **(E)** H_2_O_2_ and **(F)** ROS after 24 h culture. H_2_O_2_ was measured by hydrogen peroxide test kit, and ROS level was assessed using the probe 2′,7′-dichlorodihydrofluorescein diacetate (DCFH-DA) following TFP5 treatment. **(G,H)** The change of the mRNA level of IL-6 **(G)** and NFKB **(H)** expression in MPC-5 under HG culture condition. The cells were treated for 24 h under LG and HG conditions. GAPDH was regarded as the reference gene. **(I–K).** The mRNA level of Bax **(I)**, Bcl-2 **(J)** and caspase 3 **(K)** under HG treatment in MPC-5 cells. The cells were treated for 24 h under LG and HG conditions. GAPDH was regarded as the reference gene. **(L)** The effect of TFP5 treatment on the HG-induced apoptosis in MPC-5 cell lines. Propidium Iodide (PI) was used to separate the viable cells, and Annexin-FITC was used to identify the apoptotic cells. *p < 0.05, **p < 0.01. A *p* value less than 0.05 was considered as statistically significant.

To investigate the effects of HG treatment on CDK5 expression and activity, we cultured the mouse podocytes MPC-5 cells, respectively, under LG and HG conditions at multiple timepoints. We found that MPC-5 cells treated with HG exhibited higher level of CDK5 expression in transcriptional and translational levels compared with that under LG (Figures 1A,B). Consistently, we also detected the higher CDK5 expression under HG by flow cytometry (Figure 1C; Supplementary Figure S1). Furthermore, we measured the effect of HG on CDK5 activity, and we found CDK5 activity significantly increased under HG culture conditions (Figure 1D). Taken together, HG upregulates CDK5 activity and expression levels in mouse podocytes.

### HG Leads to High Oxidative Stress, Inflammatory Cytokines, and Apoptosis of Podocytes

Previous studies have closely correlated CDK5 activity with oxidative stress-induced cell death (Sahlgren et al., 2006; Guo et al., 2018). To investigate the influence of HG treatment on the properties of MPC-5 cells, we conducted the detection of the oxidative stress, and apoptosis-related genes in MPC-5 cells under HG condition. HG treatment significantly upregulated the levels of H_2_O_2_ and ROS compared to that under LG condition, indicating the enhanced oxidative stress after HG treatment (Figures 1E,F). Furthermore, IL-6 and NFKB, two important inflammatory cytokines, exhibited obvious increases after 48 h under HG condition (Figures 1G,H). Finally, HG led to higher Bax and caspase 3 and decreased Bcl-2 level (Figures 1I–K). Consistently, the apoptosis staining with PI and Annexin assay exhibited the higher MPC-5 apoptosis under HG conditions (Figure 1L). Therefore, the increased expression level of CDK5 induced by HG may be correlated with podocyte injury in diabetes.

### TFP5-Mediated CDK5 Activity Suppression Reduced HG-Induced Oxidative Stress, Inflammatory Cytokines, and Podocyte Apoptosis

**FIGURE 2:**
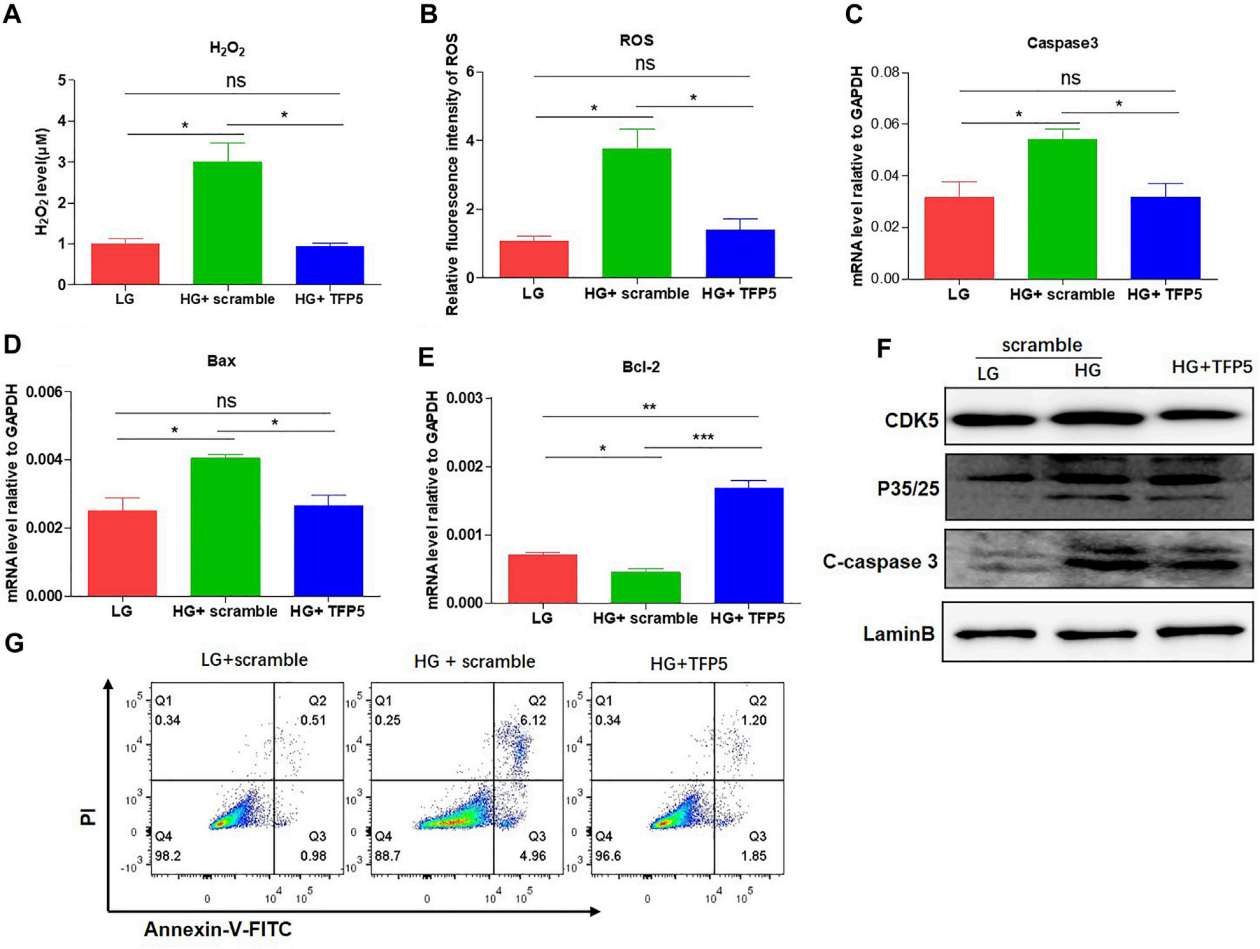
TFP5-mediated CDK5 suppression reduces HG-induced oxidative stress, inflammatory cytokines, and apoptosis in podocytes. **(A)** The effect of TFP5 on the level of H_2_O_2_ under 24 h HG treatment. H_2_O_2_ was measured by hydrogen peroxide test kit. **(B)** The detection of ROS after TFP5 treatment. ROS Level was assessed using the probe 2′,7′-dichlorodihydrofluorescein diacetate (DCFH-DA) following TFP5 treatment. **(C–E)** The effect of TFP5 of the mRNA level of **(A)** H_2_O_2_, **(B)** ROS, **(C)** Caspase-3, **(D)** Bax, and **(E)** Bcl-2 expression of MPC-5 after 24 h HG treatment. **(F)** The effect of TFP5 on the protein level of CDK5, p35/25 and apoptosis-related cleaved caspase-3 in MPC-5 after 24 h HG treatment. **(G)** The effect of TFP5 on HG-induced apoptosis of MPC-5 cells. PI was used to separate the viable cells and Annexin-FITC was used to identify the apoptotic cells. *p < 0.05, **p < 0.01, ***p < 0.001. A *p* value less than 0.05 was considered as statistically significant.

To demonstrate whether CDK5 is necessary for the podocyte injury under HG, we suppressed CDK5 activity and expression by TFP5, a truncated 24 amino acid peptide derived from the p35 protein. TFP5 significantly decreased the level of H_2_O_2_ (Figure 2A) and ROS (Figure 2B) level, indicating the reduced oxidative stress in MPC-5 cells after TFP5 treatment under HG treatment. In addition, we also detected the decreased cleaved-caspase-3 (Figure 2C) and Bax (Figure 2D) and increased Bcl-2 (Figure 2E) level after TFP5 treatment, indicating the decreased apoptotic MPC-5 cell after TFP5 treatment under HG. The western blot assay showed that HG significantly increased the levels of P35, P25, and C-caspase 3, and TFP5 treatment decreased the levels of CDK5, p35, P25, and cleaved-caspase 3 under HG condition (Figure 2F). Furthermore, TFP5 treatment strongly inhibited MPC-5 apoptosis under HG culture condition (Figure 2G). Taken together, TFP5 may be a novel CDK5 inhibitor, and attenuates podocytes injury under HG condition.

### HG Altered the Transcriptome and Suppresses Sirt1 and NGF Levels in Mouse Podocytes

**FIGURE 3:**
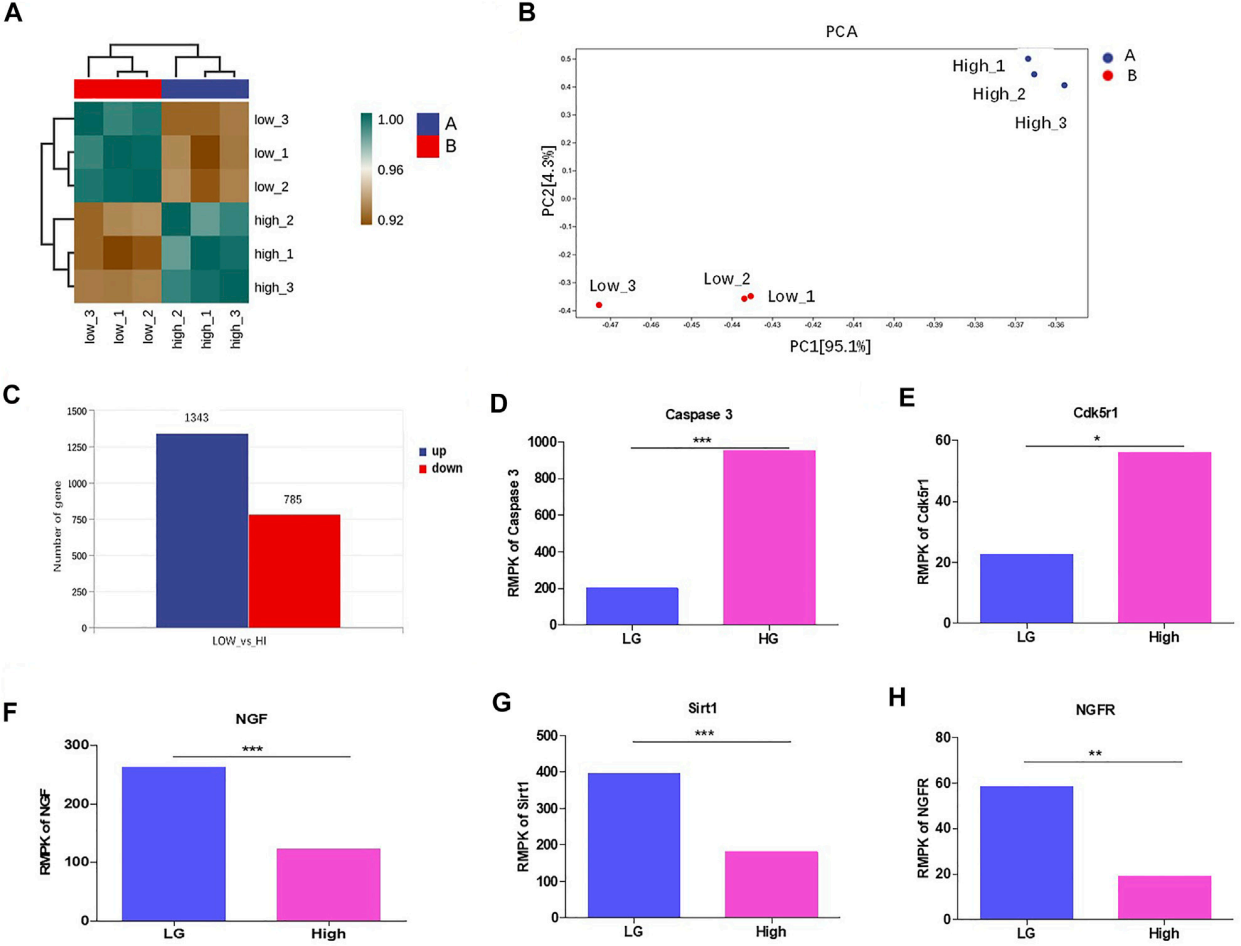
HG altered the transcriptome and suppressed Sirt1 and NGF levels in podocytes. **(A)** The correlation analysis between MPC-5 cells treated with 24 h HG and LG treatments. *n* = 3. The clustering analyses were evaluated by the gene expression condition of mRNA level. **(B)** PCA analysis of MPC-5 cells treated under HG and LG conditions *n* = 3. **(C)** The number of differentially expressed genes between HG and LG groups. The threshold was set as the fold change ≥2. **(D–H)** The effect of 24 h HG treatment on the mRNA level of **(D)** caspase-3 **(E)** Cdk5r, **(F)** NGF, **(G)** Sirt1, **(H)** NGFR. FPKM: fragments per kilobase of transcript per Million reads. *p < 0.05, **p < 0.01. A *p* value less than 0.05 was considered as statistically significant.

To study the underlying mechanism, we performed RNA sequencing for MPC-5 cell cultured under LG and HG condition. The results showed that the three repeats in low or high group exhibited good intra-clustering. Meanwhile, the LG and HG groups have different expression panels (Figure 3A). The Principal Component Analysis (PCA) showed that LG- and HG-treated MPC-5 cells showed statistically different expression spectrums (Figure 3B). Totally, 1,343 genes increased and 785 genes decreased with the change threshold of 1.5 times compared with the LG group (Figure 3C). In the differentially expressed genes, we indeed observed the upregulation of casepases-3 (Figure 3D) and CDK5r1 (Figure 3E) under HG condition. Meanwhile, NGF, Sirt1 and NGFR were significantly decreased after HG treatment (Figures 3E,F,G). Interestingly Sirt1 is one of the most important substrates of CDK5. Taken together, HG alters the transcriptome of mouse podocytes.

### TFP5 Counteracted HG Induced Sirt1 and NGF Decrease in Mouse Podocytes

**FIGURE 4:**
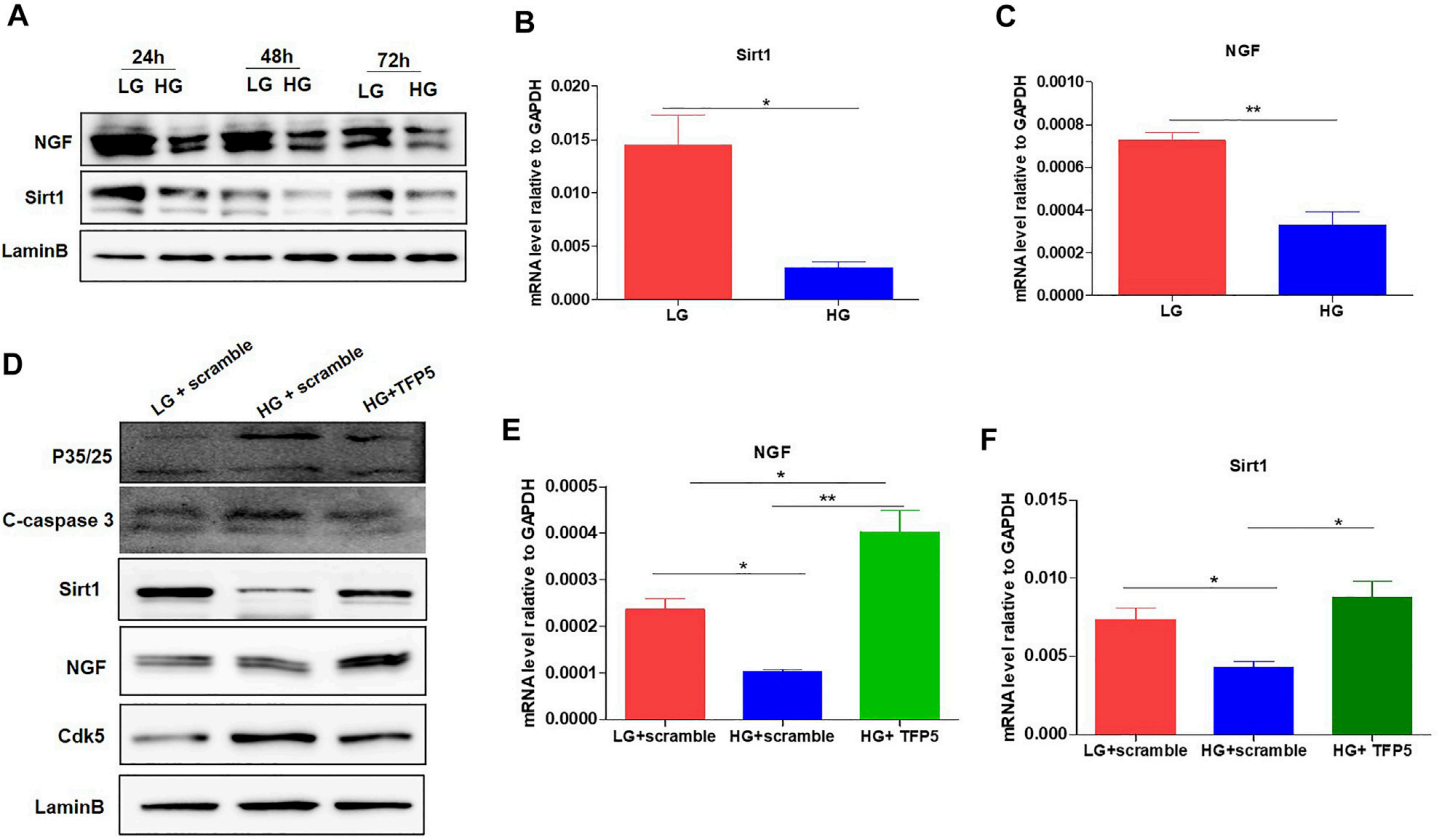
The effect of TFP5 on the oxidative stress and apoptosis resulted from HG treatment in podocytes. **(A)** The effect of HG treatment on the protein level of NGF of MPC-5 cell lines. The cells were treated for 24, 48, and 96 h, respectively. LaminB was regarded as the loading control. **(B,C)** The effect of 24 h HG treatment on the mRNA level of Sirt1 **(B)** and NGF **(C)** in MPC-5 cell lines. GAPDH was regarded as the reference gene. **(D)** The effect of TFP5 treatment on the protein level of P35/25, cleaved-caspase 3, Cdk5, NGF, and Sirt1 under 24 h LG and HG in podocytes. **(E,F)** The effect of TFP5 treatment on the mRNA level of NGF **(E)** and Sirt1 **(F)** under 24 h LG and HG in MPC-5 cell lines. *p < 0.05, **p < 0.01. A *p* value less than 0.05 was considered as statistically significant.

To confirm the correlation between TFP5 treatment and Sirt1 and NGF expressions, we detected the expression of NGF and Sirt1 after TFP5 treatment. The Western blot showed that HG decreases the expression level of NGF and Sirt1 at 24, 48, and 72 h (Figure 4A). Consistently, we observed the significant decrease of Sirt1 and NGF at transcriptional level (Figures 4B,C). TFP5 treatment significantly inhibited the expression of C-caspase-3 and CDK5 and increased Sirt1 and NGF levels (Figures 4D,E,F). Taken together, TFP5 could rescue HG-induced decrease of Sirt1 and NGF in mouse podocytes.

### NGF Overexpression Overcomes the Oxidative Stress and Apoptosis Under HG in Mouse Podocytes

**FIGURE 5:**
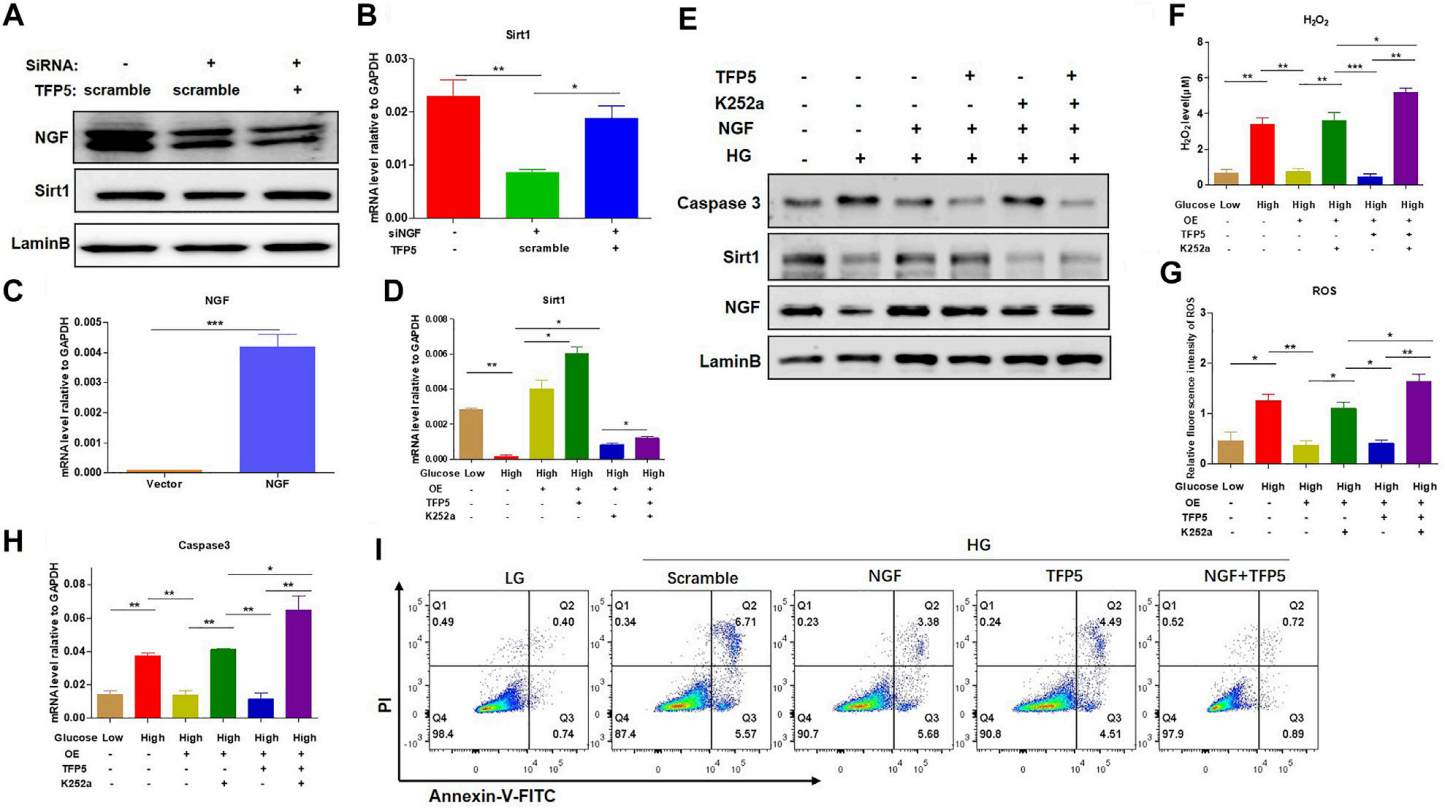
NGF overexpression overcame the HG-induced oxidative stress and apoptosis in podocytes. **(A)** The effect of NGF knockdown on Sirt1 expression level under 24 h HG treatment in mouse podocytes. LaminB was regarded as the loading control. **(B)** The effect of NGF knockdown on the mRNA level of Sirt1 and in mouse podocytes. GAPDH was regarded as the reference gene. **(C)** The verification of NGF mRNA level in NGF overexpressed MPC-5 cells. **(D)** The effect of NGF expression and TFP5 treatment on the mRNA level of Sirt1 in MPC-5 cells. **(E)** The effect of NGF expression on the protein level of caspase-3 and Sirt1 MPC-5 cells. **(F)** The effect of NGF overexpression and TFP5 treatment on the level of H_2_O_2._ **(G)** The effect of NGF overexpression and TFP5 treatment on the level ROS. ROS level was assessed using the probe 2′,7′-dichlorodihydrofluorescein diacetate (DCFH-DA) following TFP5 treatment. **(H)** The effect of NGF overexpression on the mRNA level of caspase-3 after 24 h HG treatment. **(I)** The detection of MPC-5 cells apoptosis under NGF overexpression and TFP5 treatments by C-flow cytometry. PI was used to separate the viable cells and Annexin-FITC was used to identify the apoptotic cells. *p < 0.05, **p < 0.01, ***p < 0.001. A *p* value less than 0.05 was considered as statistically significant.

To identify the necessity of NGF in HG-induced podocytes apoptosis, we knockdown NGF by siRNA. We found that NGF knockdown significantly decreased the mRNA and protein level of Sirt1 (Figures 5A,B). Furthermore, TFP5 treatment-rescued NGF knockdown induced Sirt1 decrease (Figures 5A,B). On the other hand, we overexpressed NGF in MPC-5 cell (Figure 5C). We found that NGF overexpression significantly upregulated Sirt1 expression level and under HG condition (Figures 5D,E). Furthermore, the combination of TFP5 and NGF overexpression exhibited even higher Sirt1 expression than NGF overexpression alone under HG treatment (Figures 5D,E). k252a, an NGF-specific inhibitor, significantly decreased Sirt1 expression in TFP5 treatment and NGF overexpressed MPC-5 under HG condition, indicating the dominant role of NGF on the regulation of Sirt1. Meanwhile, we found that NGF overexpression and TFP5 treatment decreased the expression level of cleaved caspase-3 which was upregulated by K252a treatment (Figures 5D,E). In addition, we also observed the obvious decrease of H_2_O_2_ and ROS after NGF overexpression, and k252a treatment counteracted the function of NGF overexpression and TFP5 treatment (Figures 5F,G). Finally, we found that NGF overexpression inhibited HG-induced MPC-5 cell apoptosis, and TFP5 exhibited the synergistic effect with NGF overexpression (Figures 5H,I). In conclusion, NGF plays an important role in HG-induced MPC-5 apoptosis.

### NGF Inhibitor Undermines the Function of TFP5 on Diabetic Nephropathy in *db/db* Mouse Model

**FIGURE 6:**
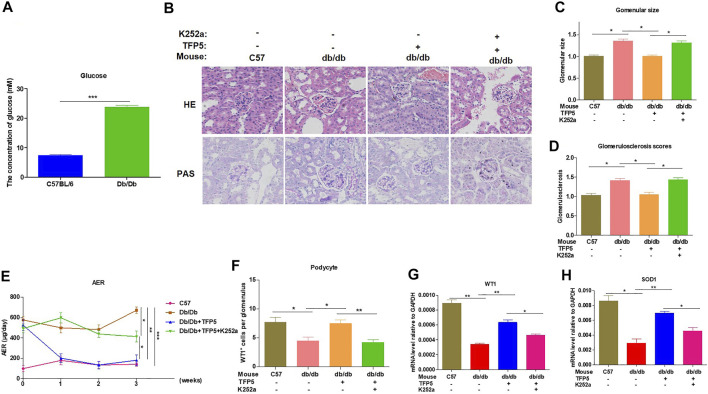
TFP5 treatment alleviated renal function in *db/db* mouse with diabetic nephropathy. **(A)** The detection of serum glucose level in C57/BL6J and *db/db* mouse model, *n* = 5. **(B)** HE staining and PAS staining were performed in all four groups. The C57BL/6J mouse was used as control group. C57BL/6J, *n* = 5; *db/db*, *n* = 5; *db/db* + TFP5, *n* = 5; *db/db* + TFP5 + K252a, *n* = 5. **(C)** The glomerular size was measured and analyzed for all four groups. C57BL/6J, *n* = 5; *db/db*, *n* = 5; *db/db* + TFP5, *n* = 5; *db/db* + TFP5 + K252a, *n* = 5. **(D)** The glomerulosclerosis score was evaluated in all four groups. C57BL/6J, *n* = 5; *db/db*, *n* = 5; *db/db* + TFP5, *n* = 5; *db/db* + TFP5 + K252a, *n* = 5. **(E)** The detection of urinary albumin excretion rate (AER) over 24 h in all four groups. C57BL/6J, *n* = 5; *db/db*, *n* = 5; *db/db* + TFP5, *n* = 5; *db/db* + TFP5 + K252a, *n* = 5. **(F)** The counting of renal podocyte number in four groups. WT1 was used as the positive marker of podocytes by IHC staining. C57BL/6J, *n* = 4; *db/db*, *n* = 4; *db/db* + TFP5, *n* = 4; *db/db* + TFP5 + K252a, *n* = 4. **(G)** The mRNA level of WT1 gene in kidney. C57BL/6J, *n* = 4; *db/db*, *n* = 4; *db/db* + TFP5, *n* = 4; *db/db* + TFP5 + K252a, *n* = 4. **(H)** The mRNA level of SOD1 gene in the kidney of four groups. C57BL/6J, *n* = 4; *db/db*, *n* = 4; *db/db* + TFP5, *n* = 4; *db/db* + TFP5 + K252a, *n* = 4. *p < 0.05, **p < 0.01. A *p* value less than 0.05 was considered as statistically significant.

**FIGURE 7:**
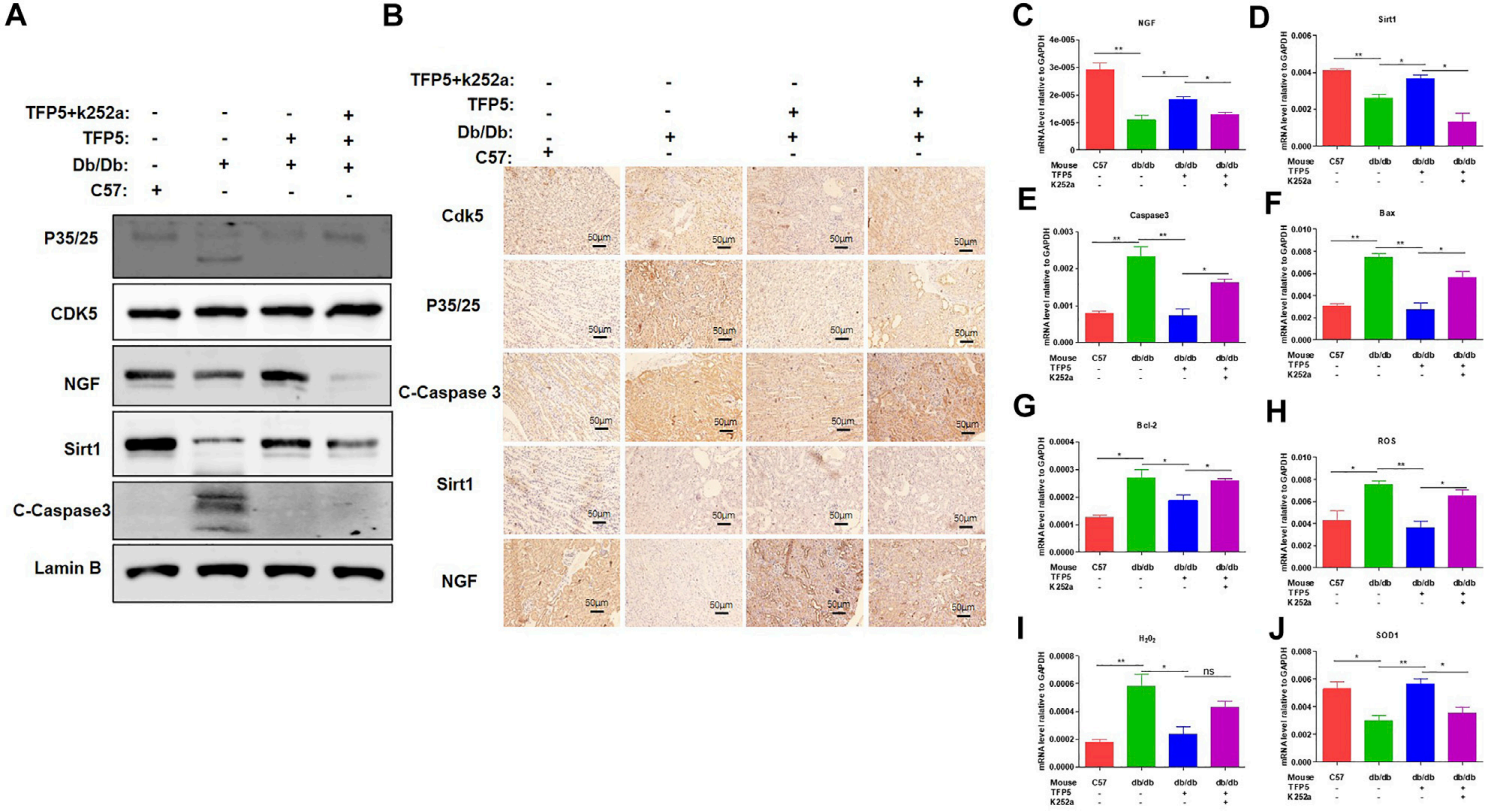
NGF inhibitor undermined the function of TFP5 on diabetic nephropathy in *db/db* mouse model. **(A)** The level of P35/25, CDK5, NGF and Sirt1 in kidney tissue after TFP5 treatment or NGF inhibition by Western blot. **(B)** IHC staining of P35/25, CDK5, NGF and Sirt1 in kidney tissue after TFP5 treatment or NGF inhibition. **(C–J)** The mRNA level of **(C)** NGF, **(D)** Sirt1, **(E)** caspase 3, **(F)** Bax, **(G)** Bcl-2, **(H)** ROS, **(I)** H_2_O_2_, **(J)** SOD1 in kidney tissue after TFP5 treatment, or NGF inhibition, or both. *p < 0.05, **p < 0.01. A *p* value less than 0.05 was considered as statistically significant.

To identify the effect of TFP5 and NGF on renal function damage caused by hyperglycemia in *db/db* mouse model, we treated mice with TFP5 peptides, K252a, or both. Firstly, we observed that the *db/db* mice showed a higher level of glucose level in serum compared to the control group (Figure 6A). As TFP5 and K252a had a profound effect on podocytes survival, we subsequently explored its influence upon glomerulus using histological staining. HE and Periodic acid–Schiff staining of kidney showed that hyperglycemia increased the glomerular size and glomerulosclerosis scores (Figures 6B–D). TFP5 treatment efficiently decreased the glomerular size and glomerulosclerosis scores which was upregulated by K252a treatment (Figures 6B–D). We also measured the urinary albumin excretion rate (AER) during the experiment. We found that hyperglycemia led to increased AER while TFP5 treatment led to decreased AER in *db/db* mouse. In addition, K252a reversed the function of TFP5 and led to severe AER (Figure 6E). In addition, we counted the number of podocytes under different treatments. We observed the decreased podocytes and TFP5 counteract hyperglycemia-induced podocytopenia (Figure 6F). Meanwhile, we used the podocytes-specific marker, WT1, by IHC assay. Consistently, WT1 positive podocytes decreased under hyperglycemia, which increased after TFP5 treatment (Figure 6G). We also measured the oxidative stress in kidney under different treatments. The results showed that the SOD1 decreased under hyperglycemia in *db/db* mouse (Figure 6H). TFP5 treatment upregulated SOD1, which was decreased by K252a treatment (Figure 6H). In addition, we also detected the expression level of P35/25, NGF, Sirt1, apoptosis and oxidative stress-related gene in the tissue after TFP5 or K252a treatment. We found that TFP5 treatment obviously decreased apoptosis (Figures 7A,B,E–G; Supplementary Figure S2) and oxidative stress- (Figures 7H,I) related genes while significantly upregulated NGF (Figures 7A–C; Supplementary Figure S2), Sirt1(Figures 7A,B,D; Supplementary Figure S2), Bcl-2 (Figure 7G) and SOD1 (Figure 7J) level in the kidney tissue of *db/db* mouse, which was undermined by k252a treatment (Figures 7A–J). In conclusion, NGF inhibitor undermines the function of TFP5 on DN in *db/db* mouse model.

### TFP5 Improved the Inflammatory Cytokines Under Hyperglycemia

**FIGURE 8:**
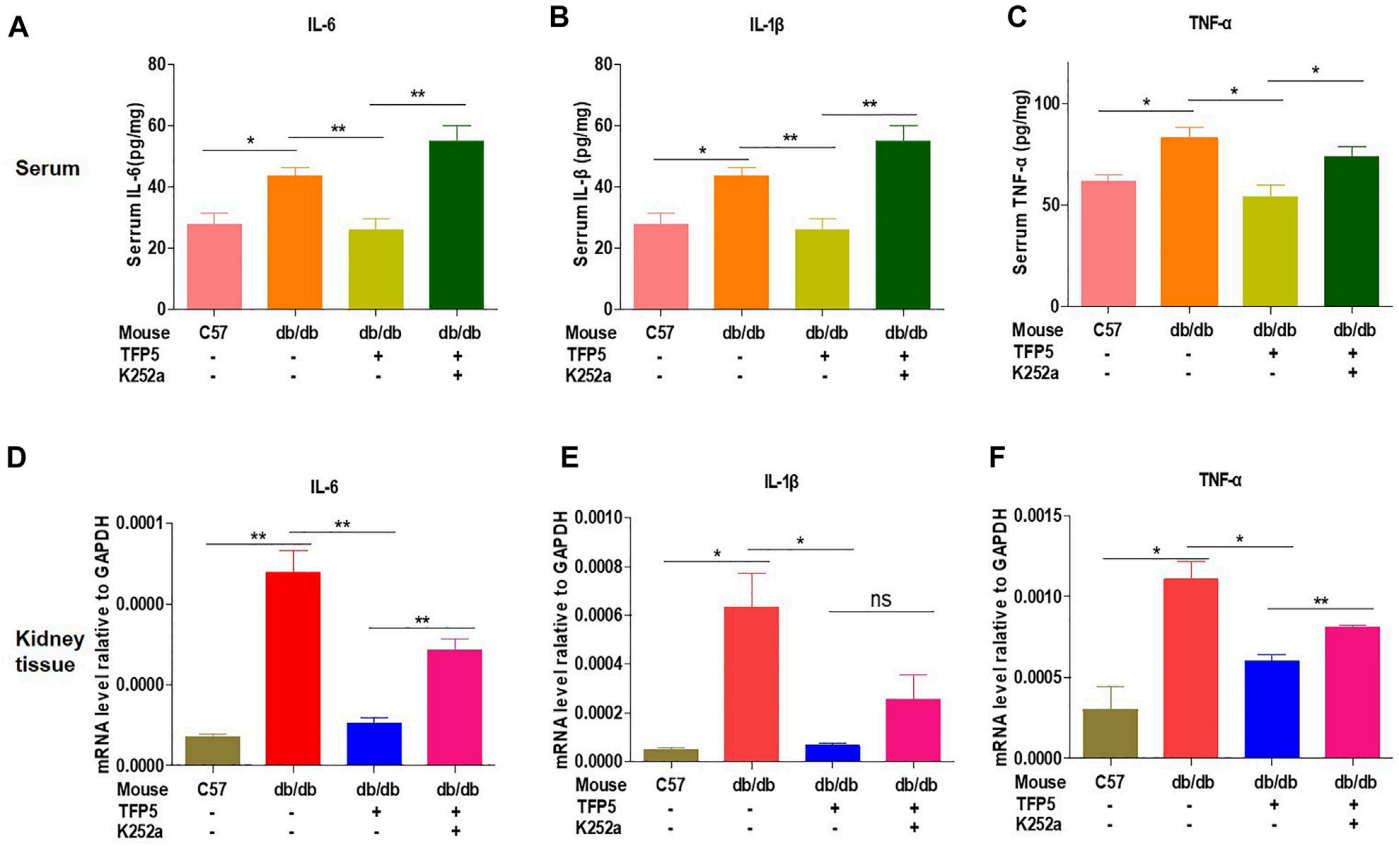
TFP5 treatment decreased the level of proinflammatory factor levels in *db/db* mouse. **(A–C)** The detection of the level of IL-6, **(A)** IL1-β, **(B)** TNFα **(C)** by Elisa in the plasma of *db/db* mice treated with TFP5, or K252a, or both. C57BL/6J, *n* = 4; *db/db*, *n* = 4; *db/db* + TFP5, *n* = 4; *db/db* + TFP5 + K252a, *n* = 4. **(D–F)** The detection of mRNA level of IL-6, **(D)** IL1-β, **(E)** TNFα **(F)** by RT-PCR in the kidney tissue of *db/db* mice treated with TFP5 or K252a or both. C57BL/6J, *n* = 3; *db/db*, *n* = 3; *db/db* + TFP5, *n* = 3; *db/db* + TFP5 + K252a, *n* = 3. *p < 0.05, **p < 0.01. A *p* value less than 0.05 was considered as statistically significant.

To investigated the effect of TFP5 on the inflammatory cytokines in serum and kidney of *db/db* mice. We found that TFP5 significantly upregulated IL-6, IL-1β, and tumor necrosis factor (TNF-α) in serum (Figures 8A–C) and kidney tissue (Figures 8D–F). TFP5 treatment decreased hyperglycemia-induced IL-6, IL-1β, and TNF-α upregulation. Furthermore, K252a treatment counteracted the effect of TFP5 and led to the upregulation of IL-6, IL-1β, and TNF-α in the serum and kidney of *db/db* model mice.

## Discussion

DN is a major long-term complication, which affects about 30% of patients with T1D, and 40% of patients with T1D (Alicic et al., 2017), is the major cause of kidney failure worldwide and the single strongest predictor of mortality in diabetic patients. Today, about 40% of patients need renal replacement therapy (Ritz et al., 2011). These classic descriptions indicate that patients with T1D develop DN within 10 years after the onset of diabetes.

Previous evidence has shown that strict glycemic control could improve the progression of DN. Although DN is considered to be a microvascular complication of diabetes, there is increasing evidence that podocyte loss and epithelial dysfunction play an important role. White et al. (2002) demonstrated that although mesangial dilatation and GBM thickening are the most common DN lesions, careful cell analysis using samples from patients with T1D and T2D showed that the number of podocytes is highly correlated with proteinuria and seems to be one of the best predictors of disease. Podocyte loss may be due to apoptosis or abscission of podocytes caused by hyperglycemia-induced ROS production (Susztak et al., 2006), indicating podocytes may be the “weakest link” in DN development. Here, we found that MPC-5 cells presented obvious apoptosis induced by oxidative stress under HG condition. TFP5, a peptide-inhibiting CDK5, efficiently inhibited HG-induced podocytes cells apoptosis and DN in the mouse model by decreasing NGF level and upregulating Sirt1 level. Therefore, our work illuminated a novel and targetable TFP5-CDK5-NGF-Sirt1 regulating axis, which may be the promising targets for diabetic kidney disease therapy in future.

TFP5 may be a promising oligopeptide drugs for diabetic kidney disease. Our previous study showed depletion of CDK5 causes significant depressed expression of WT1 and apoptosis of podocytes. Reducing ciliary body length by drug or gene inhibition of CDK5 can reduce polycystic kidney disease in renal tuberculosis model (Husson et al., 2016). Guevara et al. (2014) found that CDK5 and its regulator p35/P25 and cyclin I were also expressed in renal tubular cells. We show that treatment with CDK5 inhibitors can promote the formation of survival-promoting CDK5/cyclin I complex and improve cell survival in the case of ischemia-reperfusion Pro apoptotic injury, support the benefits of renal preservation treated with CDK5 inhibitors, and contribute to renal tubular protection. Although we previously reported that CDK5 deletion resulted in decreased WT1 expression and podocyte apoptosis, the CDK5 activity down regulated by p35 had no effect on the expression of cleaved caspase 3. On the contrary, increased apoptosis can be detected in p35 deregulated podocytes by TUNEL analysis and immunofluorescence staining of cleaved caspase 3 antibody. Podocyte viability of CDK5 and p35 knockout cells decreased (Zheng et al., 2016). In addition, Liu, 2013. reported that intermediate filament protein nestin is related to the occurrence of DN. Blocking CDK5 can increase nestin level and reduce renal damage, which will provide a useful target for the treatment of DN, indicating the crucial role of CDK5 activity on the treatment of diabetic kidney disease. In this study, we found that TFP5 effectively rescued HG-induced MPC-5 cells apoptosis and oxidative stress. In hyperglycemia-caused kidney function damage of *db/db* mouse model, TFP5 treatment increased the number of podocytes and improved the kidney function indexes.

TFP5 may regulate kidney function by effect of Sirt1 activity. Zhang et al. (2018) reported that CDK was also involved in the ubiquitin-proteasome pathway-mediated degradation of Sirt1 expression in Parkinson’s disease models, which could be efficiently blocked by the inhibition of CDK5. The phosphorylation of S47 is closely related to the nuclear retention of Sirt1, but not to telomere repeat binding factor 2-interacting protein 1. CDK5 was identified as a Sirt1 kinase that regulates s47 phosphorylation. Knockout, or inhibition of CDK5, can reduce the number of aging endothelial cells, promote the nuclear output of Sirt1, and reduce the expression of inflammatory genes in porcine aortic endothelial cells, accompanied by the accumulation of truncated regulatory subunits of CDK5, and P25 in aging porcine aortic endothelial cells and active arteries of atherosclerosis. Long-term treatment with Roscovitine blocked the development of cell senescence and atherosclerosis in the aorta of hypercholesterolemic apolipoprotein E-deficient mice (Bai et al., 2012). *In vivo* and *in vitro*, inhibition of CDK5 can reverse sevoflurane-induced neuronal apoptosis, while inhibition of CDK5 activity can promote the expression of Sirt1, which plays an important role in inducing autophagy activation. Yang et al. (2020) found that Sirt1 inhibition inhibited the protective effect of ROSC on sevoflurane-induced nerve injury by inhibiting autophagy activation. In addition, CDK5 is responsible for the phosphorylation of Sirt1 on serine 47 residue. This modification blocks the anti-aging activity of Sirt1 and plays a key role in the loss of Sirt1 function during vascular aging. Therefore, Sirt1 function can be improved by inhibiting CDK5 so as to prevent the development of atherosclerosis and slow down the process of vascular aging (Bai et al., 2012; Bai et al., 2014). Conversely, Sirt1 also regulates the expression of CDK5 through epigenetic modification. Acetylation of CDK5 at k33 (ac-CDK5) resulted in loss of ATP binding and impaired kinase activity. We identified GCN5 and Sirt1 as key factors controlling ac-CDK5 levels. Ac-CDK5 reached the lowest level in rat fetal brain, but increased significantly after birth. Interestingly, nuclear ac-CDK5 levels were negatively correlated with neurite length in embryonic hippocampal neurons, which was inhibited by Sirt1 inhibitor EX527 or acetyl simulant. The ectopic expression of (K33Q) CDK5 is inhibited, indicating that the positive regulation of Sirt1 is positively regulating the growth of neurite through the deacetylation of nuclear CDK5 (Lee et al., 2018). Consistently, we observed the significant decrease of Sirt1 under HG condition in MPC-5 cells, which was reversed by TFP5 treatment. Therefore, TFP5 may protect podocytes and improve diabetes nephropathy *via* affecting the level of Sirt1.

**FIGURE 9:**
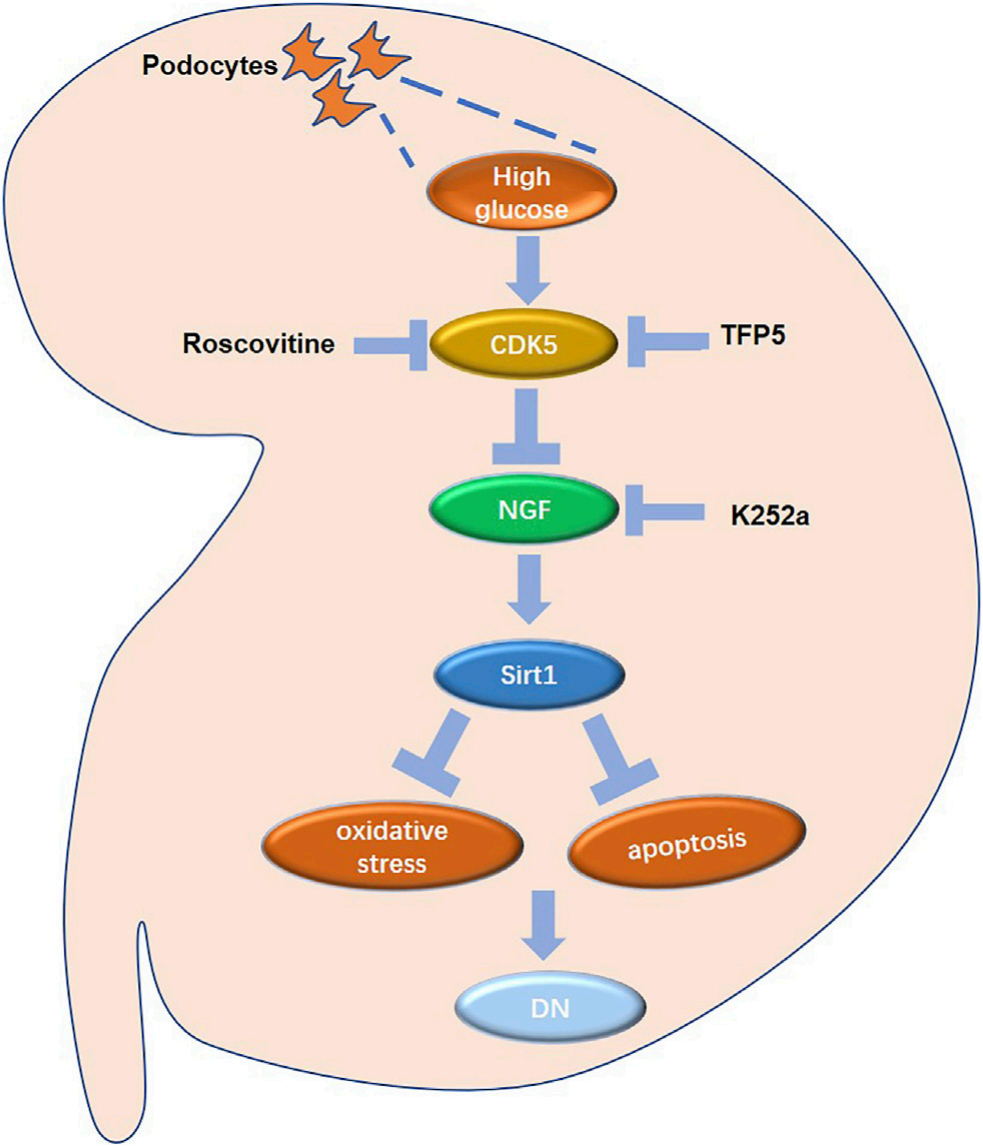
The regulatory network in mouse renal podocytes. Roscovitine: CDK5 inhibitor; TFP5: a 25 amino acid peptide that inhibiting CDK5 activity; K252a: NGF inhibitor; DN: diabetic nephropathy.

NGF may mediate the upregulation of Sirt1 after TFP5 treatment. NGF is one of the most common neurotrophic factors. It is found that neuronal apoptosis induced by the decline of NGF secretion ability of brain neuronal cells leads to neurodegenerative diseases. Previous evidence showed that during the process of apoptosis of PC12 cells (Rat neuronal cell) induced by NGF withdrawal, the activation of CDK5/p35, the protein catalyzing the phosphorylation of Sirt1, plays an important mediating role. Furthermore, Roscovitine, a specific inhibitor of CDK5, had the potential to protect neuronal cells. Apart from the regulation of the neuronal cell of NGF, Tatsuo Hata et al. reported that NGF is closely related to diabetic pathology and insulin homeostasis, indicating the potential role of NGF in hyperglycemia-induced diseases (Hata et al., 2015). Similarly, NGF withdrawal resulted in pancreatic cancer *β* Cell apoptosis. Recent studies on podocyte model *in vitro* found that podocytes, like neurons, have the ability to synthesize and secrete NGF, suggesting that NGF may be a novel target of glomerular podocytes (Caroleo et al., 2015). In our study, we found that NGF positively regulated the expression of Sirt1 in MPC-5 cells, and TFP5 significantly upregulated NGF expression. The overexpression of NGF effectively rescued HG-induced decrease of Sirt1. In addition, the k252a, the inhibitor of NGF, blocked the function of TFP5 on Sirt1 regulation (Figure 9). In conclusion, CDK5-NGF-Sirt1 axis may be a novel regulating mechanism in podocytes and *db/db* mouse models.

## Data Availability

Raw data and processed data of RNA-seq are available at the NCBI repository (SUB11139134; https://www.ncbi.nlm.nih.gov/sra/PRJNA811338).
