# Supplementary material for: TFP5-Mediated CDK5 Activity Inhibition Improves Diabetic Nephropathy via NGF/Sirt1 Regulating Axis
Source: Front Cell Dev Biol. 2022 Jul 7;10:829067. doi: 10.3389/fcell.2022.829067 (PMC9301001; doi:10.3389/fcell.2022.829067)
Supplement: Supplementary file 1 [file Table1.DOCX]

1. Flow cytometry Fcs files for Figure1C, 1L; Figure 2G, figure 5I：

<https://www.jianguoyun.com/p/De06I1wQ8t6JChiF_p4E>

1. HE stainning-All original figures for Figure 6A：

<https://www.jianguoyun.com/p/DY5WJnIQ8t6JChiV_p4E>

1. IHC stainning-All Original pictures for Figure7B：

<https://www.jianguoyun.com/p/DYR0kqUQ8t6JChiX_p4E>

1. PAS stainning-All original figures for Figure 6A：

<https://www.jianguoyun.com/p/DdBcmGgQ8t6JChiY_p4E>

1. The uncutted bands for all the western blot and alll original pictures for IHC, HE, PAS staining and flow cytometry analysis pipeline

<https://www.jianguoyun.com/p/DSrEZPsQ8t6JChig_p4E>
